# Modulating the poly-l-lysine structure through the control of the protonation–deprotonation state of l-lysine

**DOI:** 10.1038/s41598-022-24109-5

**Published:** 2022-11-16

**Authors:** Luigi Stagi, Martina Sini, Davide Carboni, Roberto Anedda, Giuliano Siligardi, Tiberiu-Marius Gianga, Rohanah Hussain, Plinio Innocenzi

**Affiliations:** 1 Department of Chemistry, Physics, Mathematics and Natural Sciences, University of Sassari, 07100 Sassari, Italy; 2 Laboratory of Materials Science and Nanotechnology (LMNT), Department of Biomedical Sciences, CR-INSTM, University of Sassari, 07100 Sassari, Italy; 3 Porto Conte Ricerche, Strada Provinciale 55, Porto Conte Capo Caccia, Km. 8,400, 07041 Alghero, SS Italy; 4 Diamond Light Source Ltd., Harwell Science and Innovation Campus, Didcot, UK; 5 College of Science, Department of Chemistry, United Arab Emirates University, Al Ain, United Arab Emirates

**Keywords:** Biochemistry, Biophysics, Chemical biology, Chemistry, Nanoscience and technology

## Abstract

Designing the architecture of l-lysine-based polymeric structures is a highly challenging task that requires careful control of the amino acid reactive groups. Conventional processes to obtain branched polylysine need several steps and the addition of specific catalysts. In the present work, to gain a better understanding and control of the formation of l-lysine-based polymers, we have investigated the correlation between the protonation state of l-lysine and the corresponding hydrothermally grown structures. The samples have been characterized by combining optical spectroscopies, such as UV–Vis, fluorescence, and synchrotron radiation circular dichroism with structural analysis by Nuclear Magnetic Resonance, Fourier Transform Infrared spectroscopy, and dynamic light scattering. We have observed that aqueous precursor solutions with alkaline pHs promote the formation of branched structures. In contrast, high pHs favour the reactivity of the ε-amino groups leading to linear structures, as shown by circular dichroism analyses. On the other hand, acidic conditions trigger the branching of the amino acid. Interestingly, the polymeric forms of l-lysine emit in the blue because the increasing number of intermolecular hydrogen bonds promote the intermolecular charge transfer responsible for the emission. Understanding the correlation between the l-lysine charged states and the polymeric structures that could form controlling the protonation–deprotonation states of the amino acid opens the route to a refined design of polypeptide systems based on l-lysine.

## Introduction

The formation of homopolypeptides from an AB_2_-type amino acid, such as l-lysine, is a complex process that can lead to several polymeric species characterized by very different chemical and physical properties. l-lysine exists in two enantiomeric forms, l and d lysine, which rotate the polarized light clockwise (D-lysine) or anticlockwise (l-lysine). A small linear hydrocarbon chain (four methylene groups) ending with a second amino group (ε), which is generally more reactive and thus accountable for the formation of different homopolypeptides, characterizes the side-chain of this amino acid. l-lysine forms linear polymers that differentiate into α-poly-l-lysine or ε-poly-l-lysine, depending on whether the polymerization occurs mostly through the nitrogen in the α or ε position^1,2^.

l-lysine, via its three reactive groups (the carboxylic acid and the amines in α and ε), can also form several branched polymeric structures, such as dendrimers, branched and hyperbranched polylysine (PLL). In general, the ordinary thermal polymerization of l-lysine produces branched PLL through amide bonding upon thermal treatment between 225 and 240 °C^3^.

Obtaining controlled branched PLL requires appropriate catalysts and protective groups to control the reactions and the final polymer in terms of topology and size^4^. On the other hand, the linear PLL may display conformational distributions with the formation of different locally organized structures, such as α-helix and β-sheets^5^.

l-Lysine is a fluorescent amino acid with a characteristic blue emission in aqueous solutions whose origin is a puzzling phenomenon. In our recent study^6^, we have demonstrated a strong correlation between photoluminescence and l-lysine charged states. l-Lysine can assume different charges as a function of pH, while the concentration controls the possible formation of aggregates. Under physiological conditions, this amino acid occurs in zwitterionic form with both the amino groups protonated, –NH^3+^, and the α-carboxylic groups deprotonated, –COO^−^. Since concentration and hydrogen-bonding interaction govern most of the chemophysical properties of amino acids in solution, understanding how they affect l-lysine polymerization is of great interest. In general, optical properties such as UV–Vis absorption and fluorescence emission are effective indicators of the l-lysine state, in terms of charge and aggregation. Furthermore, the formation of clusters of different sizes can favour intermolecular charge transfer and affect the emission phenomenon. For instance, the self-assembly of l-lysine in water at 25 °C is the phenomenon underlying the blue emission, although larger aggregates are non-fluorescent^6^.

An interesting scientific problem that has been overlooked so far is whether there is any correlation between the protonation state of l-lysine in solution and the derived polymerized structures. Establishing a correlation between the state of the l-lysine and the corresponding polymeric architecture is of great interest for fine-tuning the structure–property relationship in homo and polypeptides based on the amino acid. In this work, we have used l-lysine solutions at four different pHs, 2.5, 7.3, 9.7 and 13. We have studied how the final product changes according to the precursor conditions through a hydrothermal process (HT) at 130 and 200 °C. Hydrothermal processing is an ideal method to investigate the effects of the protonation–deprotonation states on the formation of specific polymerics species. As a result, there is a direct correlation between the initial protonation state of the l-lysine monomers and the structure and properties of the resulting polymer. In-depth knowledge of this fundamental phenomenon is of paramount interest to achieving precise control the formation and production of l-lysine-based nanostructures.

## Experimental section

### Synthesis of PLL

L-Lysine (> 98%, FG), hydrogen chloride (HCl, 37%) and sodium deuteroxide (NaOD) solutions, were purchased from Sigma Aldrich, and sodium hydroxide anhydrous pellets were purchased from Carlo Erba Reagent, ITA. Deuterium chloride (DCl, 20%) was purchased from Cambridge Isotope Laboratories, Inc. Distilled water was used as a solvent.

l-Lysine nanopolymers were obtained by hydrothermal treatment. 0.128 g of l-lysine were solubilized in 10 mL of distilled H_2_O (0.87 M). The solutions were placed into a Teflon-lined stainless steel autoclave^7^ with a capacity of 50 mL and then heated at 130 or 200 °C for 15 h. After the hydrothermal treatment (HT), the obtained products were cooled down to 20 °C and then used for the characterizations. The samples hydrothermally treated at 130 and 200 °C are indicated in the text as HT-130 °C and HT-200 °C.

The pH of the precursor aqueous solutions of l-lysine was measured with a pH meter (pH 80 + DHS, Xs instrument). Four different pH values were used for the experiments: 2.5. 7.3 (obtained by adding 1 M HCl dropwise), 9.7 and 13 (obtained by adding droplets of 1 M NaOH).

### Characterizations

UV–Vis absorption spectra were acquired by a Nicolet Evolution 300 interfaced with the Vison Pros software, in the 200–600 region.

Fluorescence measurements were performed using a Horiba Jovin Yvon Fluoromax-3 spectrometer. Photoluminescence quantum yield (QY) measurements were performed using a quanta-*φ* (HORIBA) integrating sphere accessory, connected to the Jobin Yvon "NanoLog" Horiba.

Infrared absorption spectra were recorded by Fourier Transform Infrared Spectroscopy (FTIR) with a Bruker Vertex 70 infrared interferometer, in the 4000–400 cm^−1^ region. The spectra were recorded by averaging 256 scans with a 4 cm^−1^ resolution. The measures were realized using tablets prepared by mixing the samples with KBr (99%, Sigma Aldrich). FTIR spectra of l-lysine in solution at different pDs were recorded in Attenuated Total Reflection (ATR) mode, using D_2_O as solvent. pH values have been measured by a digital pH meter (XS Instruments) and corrected for pDs according to the protocol reported in ref.^8^. In situ FTIR analysis as a function of the temperature was done employing a Hellma heating jacket to heat KBr pellets with the samples. The measurements were performed from 25 to 200 °C, with steps of 10 °C and a heating rate of 2 °C min^−1^.

Two-Dimensional FTIR Correlation has been performed based on the comparison of the spectral intensity changes as a function of the temperature (the perturbation parameter). The synchronous and asynchronous correlation maps were plotted to take the first absorption spectrum as a reference; the map was traced using OPUS 7.0 software.

All NMR spectra were acquired using a Bruker Advance instrument at 600 MHz proton frequency (Bruker BioSpin GmbH, Karlsruhe, Germany). Bruker BBI 5 mm probe with z-gradients was used. All measurements were performed at T = 298 K (Bruker BVT3000 and BCU05 temperature control units). NMR 1D ^1^H NMR and 2D ^1^H–^13^C HSQC and ^1^H–^13^C HMBC were acquired using *J*_CH_ = 145 Hz and long-range coupling optimized sequences, respectively. Approximately 10 mg of each sample were weighed and dissolved in 1 mL of a 50 mM phosphate buffer solution in D_2_O (99.9%, Cambridge Isotope Laboratories Inc., Andover, MA, USA) at pH 4.5 with added Trimethylsilylpropanoic acid (TMSP) used as a chemical shift standard (δ = 0 ppm).

Circular dichroism (CD) experiments were performed using a nitrogen-flushed Chirascan Plus CD spectropolarimeter (Applied Photophysics Ltd, Leatherhead, UK) as well as Module A of B23 beamline for synchrotron radiation circular dichroism (SRCD) (Diamond Light Source Ltd, Didcot, UK). For the CD/SRCD analysis, the samples were redissolved in water at 0.5 mg mL^−1^ concentration with l-lysine as the reference studied at the same 0.5 mg mL^−1^ concentration using a 0.1 cm pathlength with the exception of pH 13 where the pathelength was 0.01 cm. The CD spectra of Leucyl–leucine (Leu–Leu) (0.5 mg mL^−1^ in ethanediol-H_2_O (2:1) were measured with a Jasco CD spetropolarimeter J720 in the − 110 °C to + 80 °C temperature range at about 20 °C intervals^9^.

The samples were studied across near- and far-UV regions (190–350 nm) in a rectangular cuvette of 0.1 cm path length (Starna). The measurements were acquired using an integration time of 1 s, 1 nm bandwidth at 20 °C. Four consecutive spectra were scanned for the solvent (water) and 1 spectrum for the sample. The thermal melting studies were performed across 80–96 °C temperature range, with two minutes of equilibration time, and a final acquisition back at 80 °C. The SRCD photo denaturation studies were performed on B23 beamline Module A measuring 30 consecutive repeated scans for each sample of l-lysine treated at 130 °C. In the case of 200 °C treated samples, the SRCD magnitude was too small for meaningful assays. The data were processed using CDApps^10^ and OriginLab™. The α-helix, β-strand, turn and unordered conformations content of the secondary structure were estimated using CONTINN algorithm^11^ of B23 CDApps^10^.

The dynamic light scattering experiments were conducted on Zetaseizer Nano (Malvern Panaltytical) with the same solution concentrations used for CD and SRCD studies.

## Results and discussion

**Figure 1:**
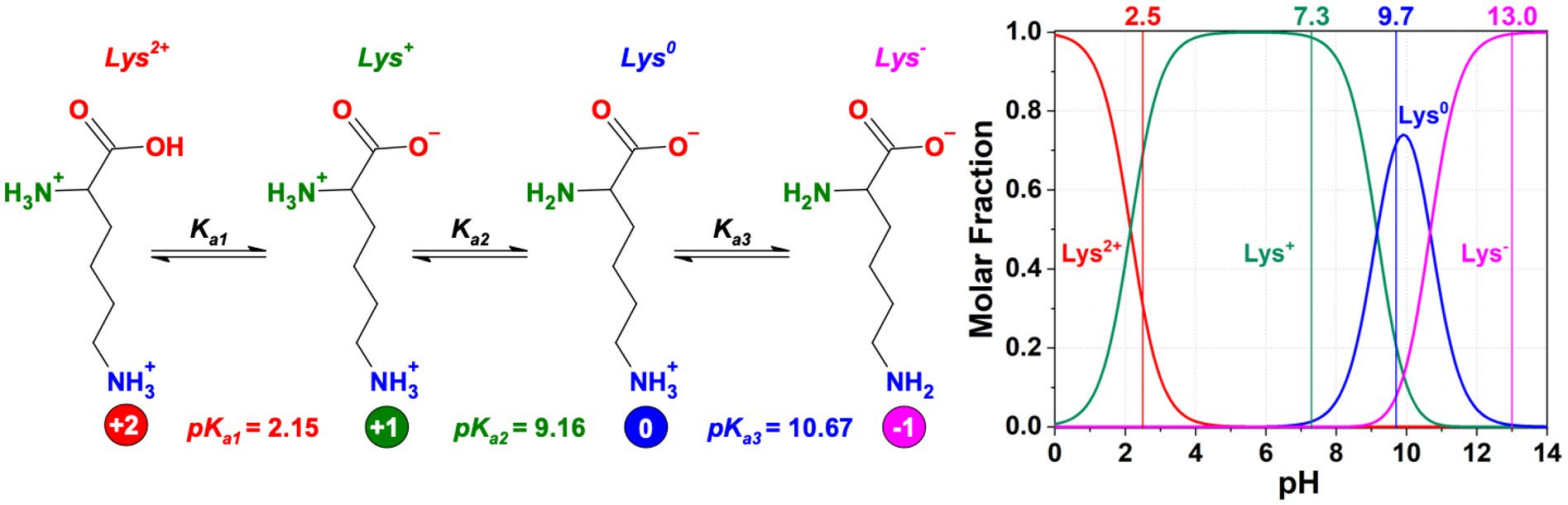
Charges of l-lysine as a function of pH. The data have been taken from references^15,16^.

L-Lysine is an asymmetrical AB_2_ amino acid^4^ highly soluble in water, up to 100 mg mL^−1^, giving a highly transparent and colourless solution. It has, however, a strong tendency to aggregate at high concentrations forming fluorescent species^12,13^. In addition to molarity, the optical properties of l-lysine depend on the pH of the solution. In aqueous solutions, l-lysine can occur as a cationic, an anionic, or zwitterionic molecule as a function of pH (Fig. 1). The net charge changes from + 2 at low pH (pH < 2.15), when the carboxylic groups and amino groups are all protonated, to + 1 in the case of deprotonation of the carboxylic and protonation of the amine groups (2.15 < pH < 10.62), to 0 when l-lysine is mainly in the zwitterionic form with the terminal ε-amino group of the aliphatic chain protonated and the carboxylic group deprotonated. It becomes negatively charged, − 1, in highly basic conditions (pH > 10.67) when the amino groups are fully neutralized and the carboxylic group is still deprotonated^14^.

The charge of l-lysine at different pHs affects the aggregation state and optical properties in the solution^3,6^. Therefore, we deduce that the pH-induced polarity must affect the formation of homopeptides and, correspondingly, originates different PLL structures.

**Figure 2:**
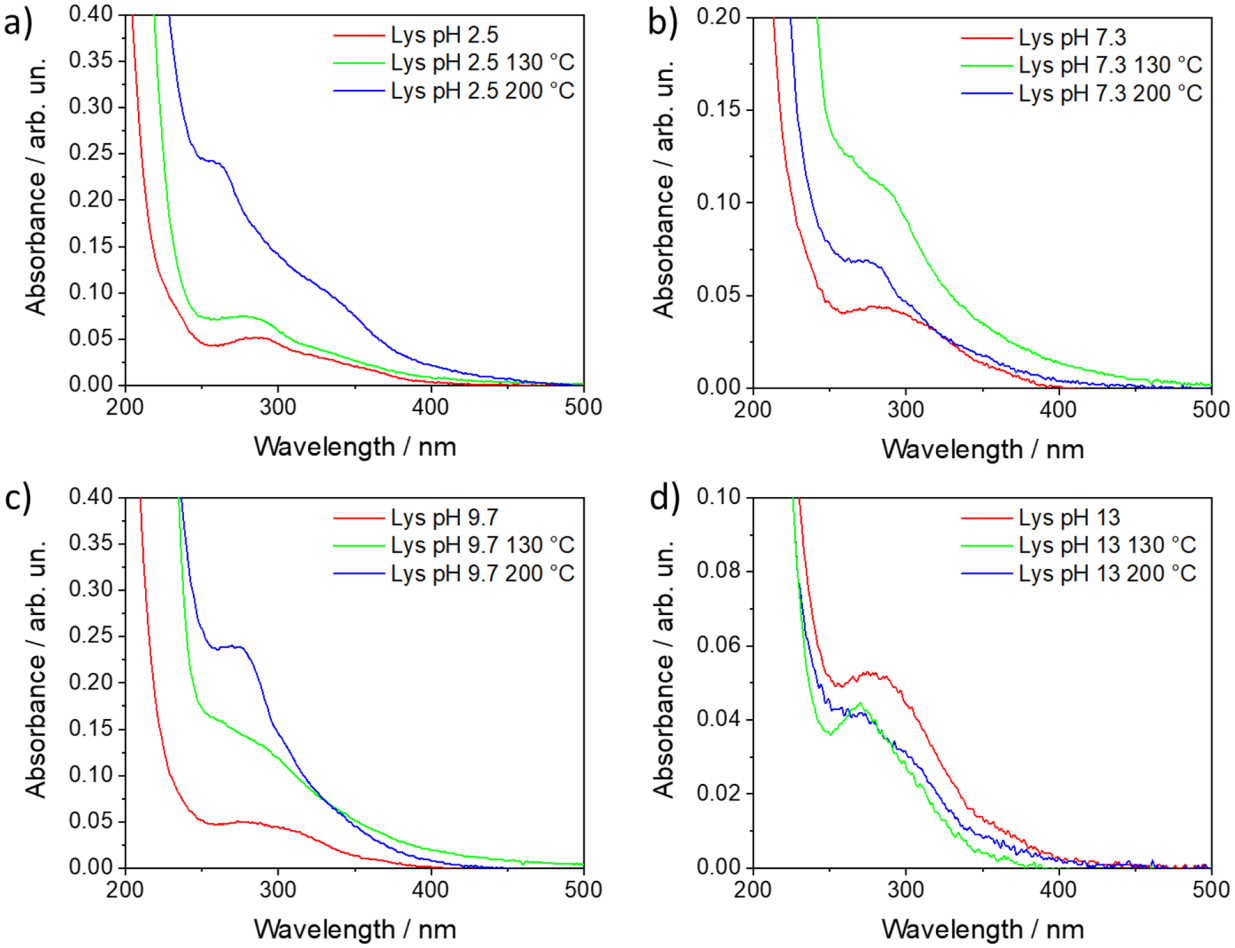
UV–Vis absorption spectra in the 200–500 nm range of l-lysine at pH 2.5 (**a**), 7.3 (**b**), 9.7 (**c**), and 13 (**d**). The red, green, and blue curves show the absorption spectra of the as-prepared aqueous solution, and after hydrothermal treatment at 130 and 200 °C, respectively. The concentration of l-lysine in the precursor solution was 0.87 M.

Figure 2 shows the UV–Vis absorption spectra of l-lysine in aqueous solutions at different pH values: 2.5, 7.3, 9.7 and 13, at 25 °C and after the HT at 130 and 200 °C. Apart from pH 2.5, where two species are present, the other pH values have been chosen to assess the reactivity of one predominant state of the l-lysine charge. At pH 2.5, we have the coexistence of the fully protonated, **Lys**^**2+**^ (about 31%), and dicationic, **Lys**^**+**^ (69%), species, while at pH 7.3, the dicationic **Lys**^**+**^ species is almost the only present (98.6%). At pH 9.7, there is a coexistence of three species, the negatively charged **Lys**^**−**^ (7%), the dicationic **Lys**^**+**^ (21%) and the zwitterionic (**Lys**^**0**^) that is predominant (72%). Finally, at pH 13, the negatively charged (**Lys**^**−**^) specie is essentially the only available (99.5%).

An intense absorption band at around 200 nm (not shown in Fig. 2), which corresponds to the fundamental (π–π*) transitions in l-lysine monomers, characterizes the UV–Vis absorption spectra of l-lysine and poly-l-lysine in water (SI1). After the hydrothermal treatment, PLL still exhibits the 200 nm band. The UV–Vis spectra also show the presence of two other overlapped absorption bands at higher wavelengths, around 275–300 and 265–310 nm. The lower energy bands are the typical signature of small hydrogen-bonded aggregates that are the source of l-lysine emissions in aqueous solutions^6^. Theoretical calculations, reported in our previous work^6^, have confirmed that l-lysine monomers absorb only at high energy (< 250 nm) with the n–π* transitions at about 230 nm. The near UV bands are due to aggregation states of this amino acid. l-Lysine monomers in aqueous solutions self-assemble into tetramer and pentamer clusters that promote intermolecular charge transfer between neighbouring species^6^. The optical properties of l-lysine in aqueous solutions depend, in fact, on the concentration governing the aggregates' formation. In addition, the pH value perturbates the l-lysine's protonation state and modulates the absorption bands' intensity and energy at higher wavelengths.

The changes in the different absorption bands as a function of the pH and processing conditions have been studied in detail by separating the different components. The absorption spectra have been deconvoluted using three components (SI2). Hydrophobic aggregation of l-lysine monomers is responsible for the absorption at around 4.5–4.3 eV (275–288 nm). This component at higher energy is always the main one, except for the sample at pH 2.5 hydrothermally treated at 200 °C. When l-lysine dissolves in water at low pHs, it is in a protonated form (Fig. 1) (fully protonated only at pH < 2.5). This charged state perturbates the aggregates by forming a different band at lower energy. After the hydrothermal treatment at 200 °C, all the samples display an additional band around 4.0–3.4 eV (310–365 nm), whose intensity and energy depend on the polymerization degree and pH of the precursor solution^6^. HT treatments at 130 °C do not produce additional absorption bands, and the systems retrieve most of the characteristic properties observed in l-lysine solutions (Fig. 2). The degree of polymerization increases at higher temperatures, and the intensity of the lowest energy band (4.0–3.4 eV) also rises in accordance. A stronger intermolecular interaction can explain this effect in correspondence of a higher number of hydrogen bonds (low pH value) and a denser structure (due to a more efficient polymerization).

**Figure 3:**
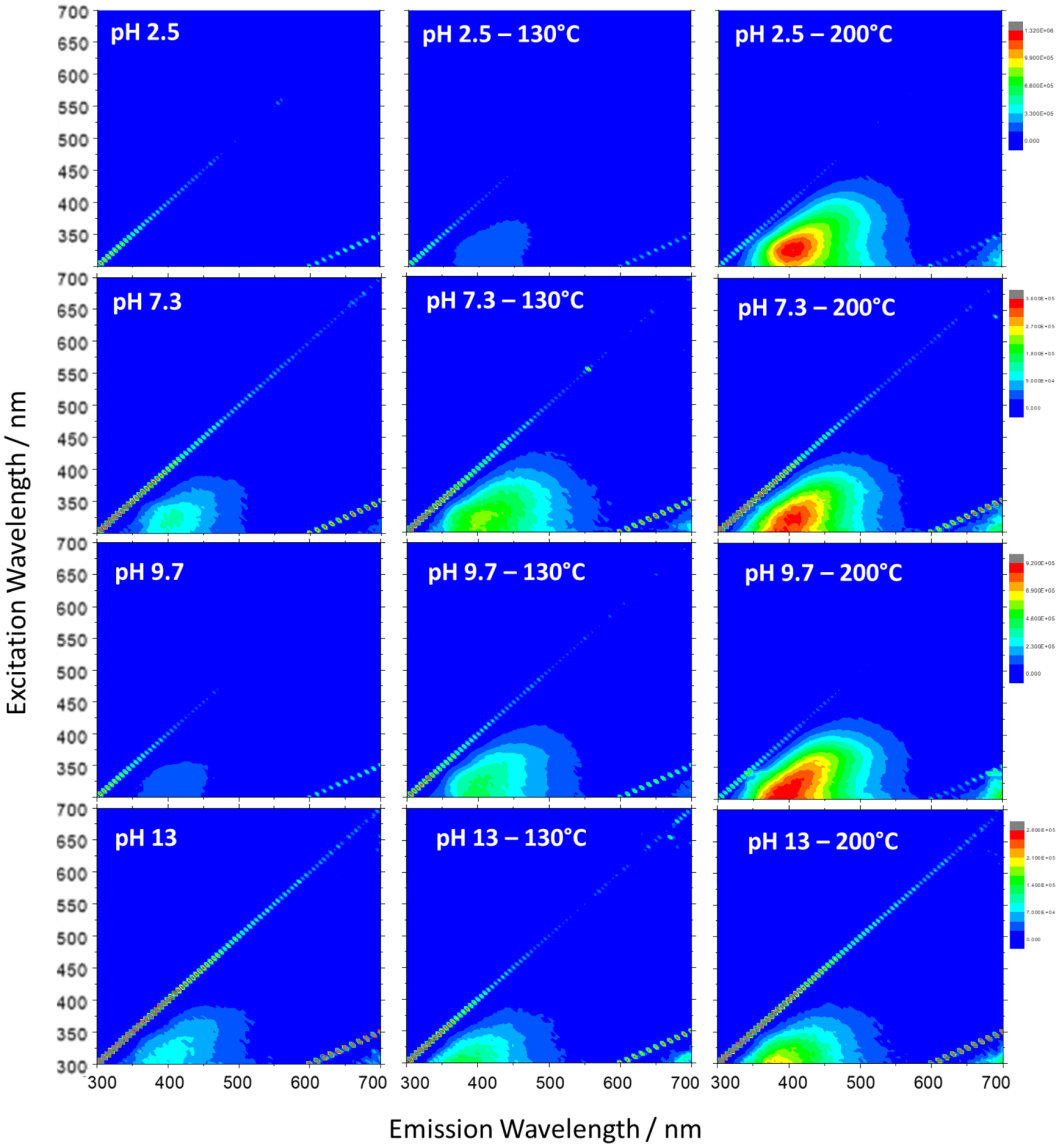
Excitation(y)–emission(x)–intensity (normalized false-color intensity scale) map of the aqueous solutions of l-lysine at different pHs and after the hydrothermal treatment at 130 and 200 °C. The concentration in the precursor solution was 0.87 M.

Figure 3 shows the 3D photoluminescence maps in the 300–700 nm range (x-emission; y-excitation; normalized false-colour intensity scale). In general, the photoluminescence of untreated l-lysine precursor at the different pHs is very weak with a minimum at pH 2.5. The emission efficiency increases for all samples as the polymerization proceeds, reaching the maximum after hydrothermal treatment at 200 °C.

Hydrothermal treatment at 200 °C of precursors that retain protonated species (pH 2.5, 7.3 and 9.7) promotes the formation of compounds interacting via analogous mechanisms as testified by a fluorescent emission peaking at around 400 nm and broadly excitable at 310–320 nm. Conversely, PLL derived by deprotonated l-lysine (pH 13) displays a weak emission at around 370 nm with excitation maximum below 300 nm. These results suggest that the emission should be connected to the protonated nature of species that are formed in the precursor aqueous solutions. Since the emission of l-lysine in solution is due to the intermolecular charge transfer through hydrogen bonding, the formation of large l-lysine structures of different types (vide infra) can enhance the extents of hydrogen bonds between the molecules. It should be at the ground of the observed emissions.

**Figure 4:**
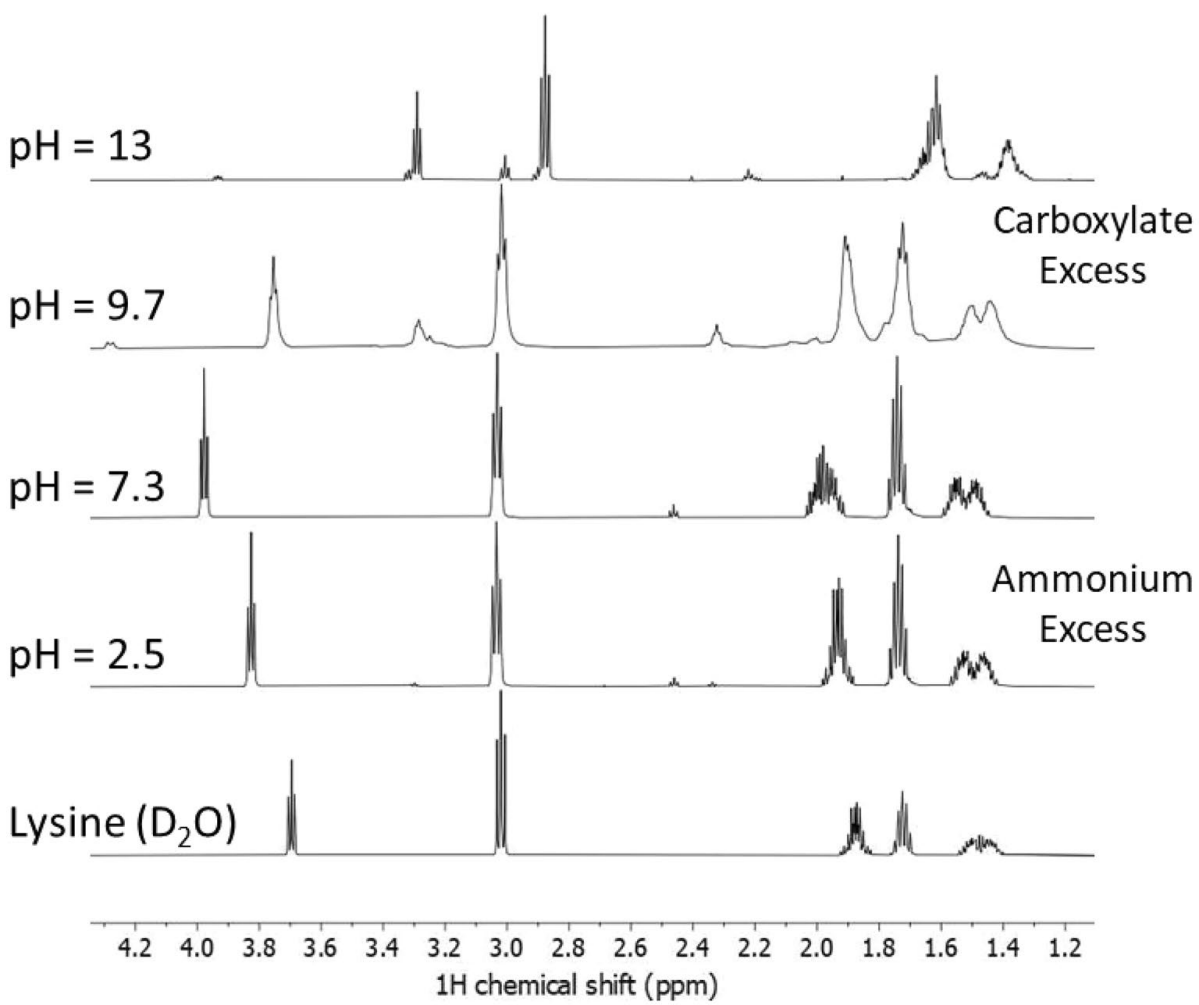
^1^H NMR spectra of the l-lysine samples after hydrothermal treatment at 130 °C using aqueous solutions at different pH. From bottom to top: untreated l-lysine and pH 2.5, 7.3, 9.7, and 13.

**Figure 5:**
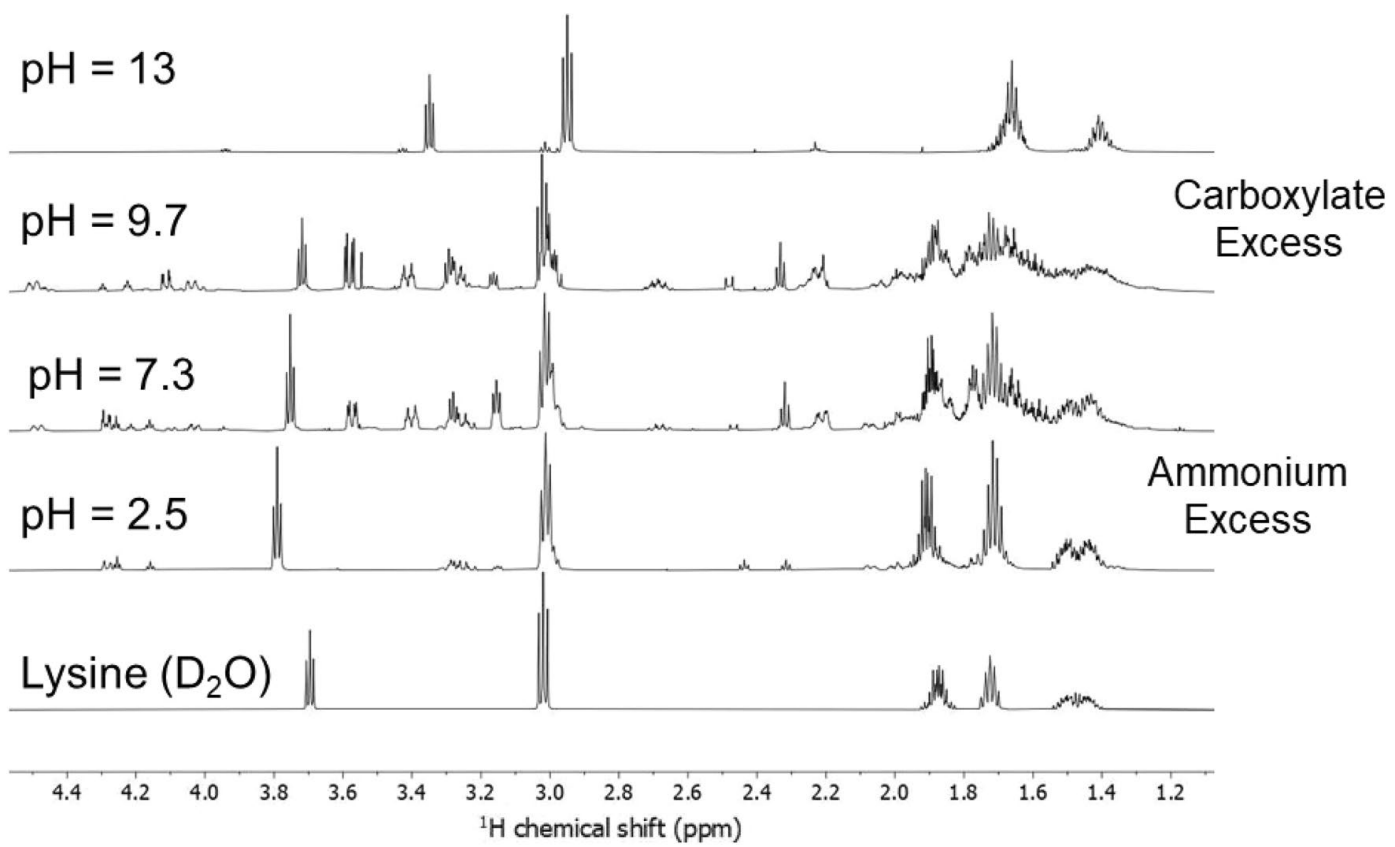
^1^H NMR spectra of the l-lysine samples after hydrothermal treatment at 200 °C using aqueous solutions at different pH. From bottom to top: untreated l-lysine and at pH 2.5, 7.3, 9.7, and 13.

The ^1^H NMR spectra of the different samples after the hydrothermal treatment (at 130 °C in Fig. 4 and at 200 °C in Fig. 5) show that the pH of the precursor aqueous solutions directly affects the structure that forms at the end of the HT process. After the hydrothermal treatment at 130 °C, NMR signals ascribed to methylene in α and β positions of l-lysine progressively shift to the low field as a function of increasing pH (Fig. 4). This shift likely reflects the effect of H-bonds formation in these systems in solution. At pH 9.7, the NMR spectrum is affected by marked line broadening, and new signals at 2.32, 3.27, 3.75, and 4.28 ppm appear. These experimental findings indicate the formation of a hyperbranched aggregate, as previously observed^17^. Detailed attributions of the different NMR chemical shifts can be found in the SI3. A further pH increase to highly basic conditions (pH 13) favors the formation of a ε-poly-l-lysine linear structure.

When the hydrothermal treatment is performed at a higher temperature (Fig. 5), 200 °C, and pH 13, the ε-poly-l-lysine linear structure still represents the main species observed by NMR. In general, thermal polymerization of l-lysine favours the formation of ε-poly-l-lysine because of the higher reactivity of ε-amine groups. The hydrothermal synthesis at pH 13 reproduces similar conditions and gives an excess of linearly linked ε-structures.

The structure is more interconnected in the intermediate range of pHs 7.3 and 9.7, showing the presence of dendrimeric and branched networks, especially recognizable by low field signals at ~ 4.5 ppm^18^. In these conditions, unreacted l-lysine and linear structures are also present (signals in the 2.9–3.8 ppm range). At low pH, the system configuration is a mix of unreacted monomers with a small number of branched polymers (4.48 ppm). The NMR characteristics of PLL resemble that of l-lysine monomer, indicating that acid conditions are more likely to loosely interconnected and not conformed polymers. These structures have a lower occurrence of intermolecular hydrogen bonds that are more common in randomly interconnected molecular arrangements.

**Figure 6:**
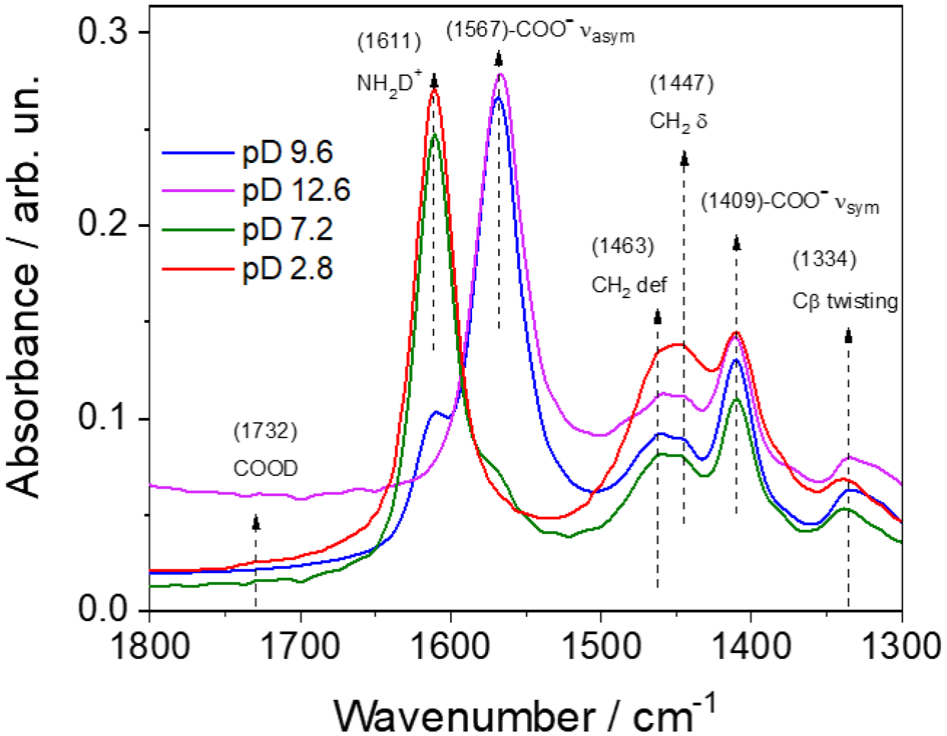
FTIR absorption spectra at 25 °C of D_2_O solutions of l-lysine at different pDs (2.8, red line; 7.2, green line; 9.6, blue line; 12.6, magenta line). The spectra have been acquired in ATR mode.

The infrared spectra of the samples well support the NMR data and indicate that the final structure is directly correlated to the protonation–deprotonation state of the precursor in aqueous solution. Figure 6 shows the FTIR spectra of l-lysine in deuterated water as a function of pH. The protonation state of l-lysine is identifiable by the modulation of specific vibrational fingerprints at different pHs^15,19^. They can be:

1. pH 2.5. About 32% of the l-lysine in solution should be in the fully protonated state (bi-cationic state): COOH (1732 cm^−1^), ε(NH_3_^+^) (δ_asym_1611 cm^−1^), α(NH_3_^+^) (δ_asym_1611 cm^−1^), (the carboxylic group and the ammonia in α and ε positions are protonated). This is the only state with the protonated carboxylic group.
2. pH 7.3. The mono-protonated species is about 98% of the l-lysine in solution (cationic state): COO^−^ (δ_asym_, 1567 cm^−1^), ε(NH_3_^+^) (δ_asym_,1611 cm^−1^), α(NH_3_^+^) (δ_asym_,1611 cm^−1^), (deformation, 1611 cm^−1^) (the carboxylic group is deprotonated and the nitrogen in α and ε positions are protonated).
3. pH 9.7. The zwitterionic species is about 73% of the l-lysine in solution (zwitterionic state): COO^−^ (δ_asym_, 1567 cm^−1^), α(NH_2_), ε(NH_3_^+^) (δ_asym_,1611 cm^−1^), (the carboxylic group is deprotonated and the ammonia in ε position is protonated).
4. pH 13. The fully deprotonated species is about 100% of the l-lysine in solution (anionic state): COO^-^ (δ_asym_, 1567 cm^−1^), ε(NH_2_), α(NH_2_) (the carboxylic group is deprotonated).

The spectrum of l-lysine at pD 2.8 (Fig. 6) is the only one to exhibit a not negligible amount of the protonated form of the carboxylic group (the bi-cationic state) and, correspondingly, the weak absorption band peaking at 1732 cm^−1^ is attributed to COOD stretching. The deprotonated state of the carboxylic groups, –COO– (νasym), is instead observed through the band at 1567 cm^−1^. This band is clearly detected only in the sample at pD 9.6.

**Figure 7:**
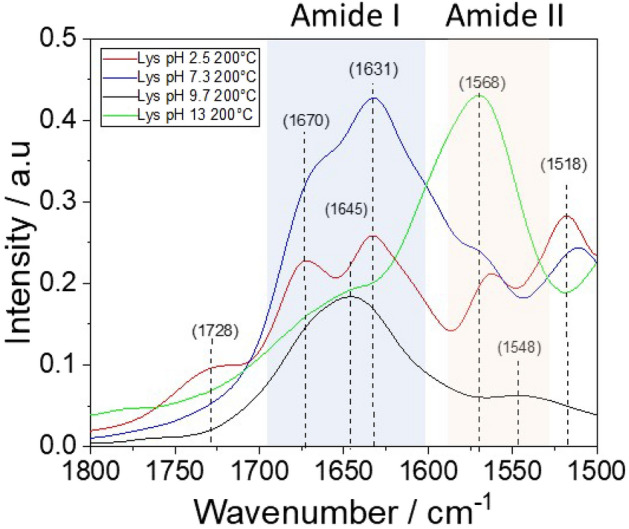
FTIR absorption spectra in the 1800–1500 cm^−1^ range of l-lysine after the hydrothermal treatment at 200 °C.

Figure 7 shows the FTIR absorption spectra in the 1800–1500 cm^−1^ range of l-lysine after the hydrothermal treatment at 200 °C.

**Figure 8:**
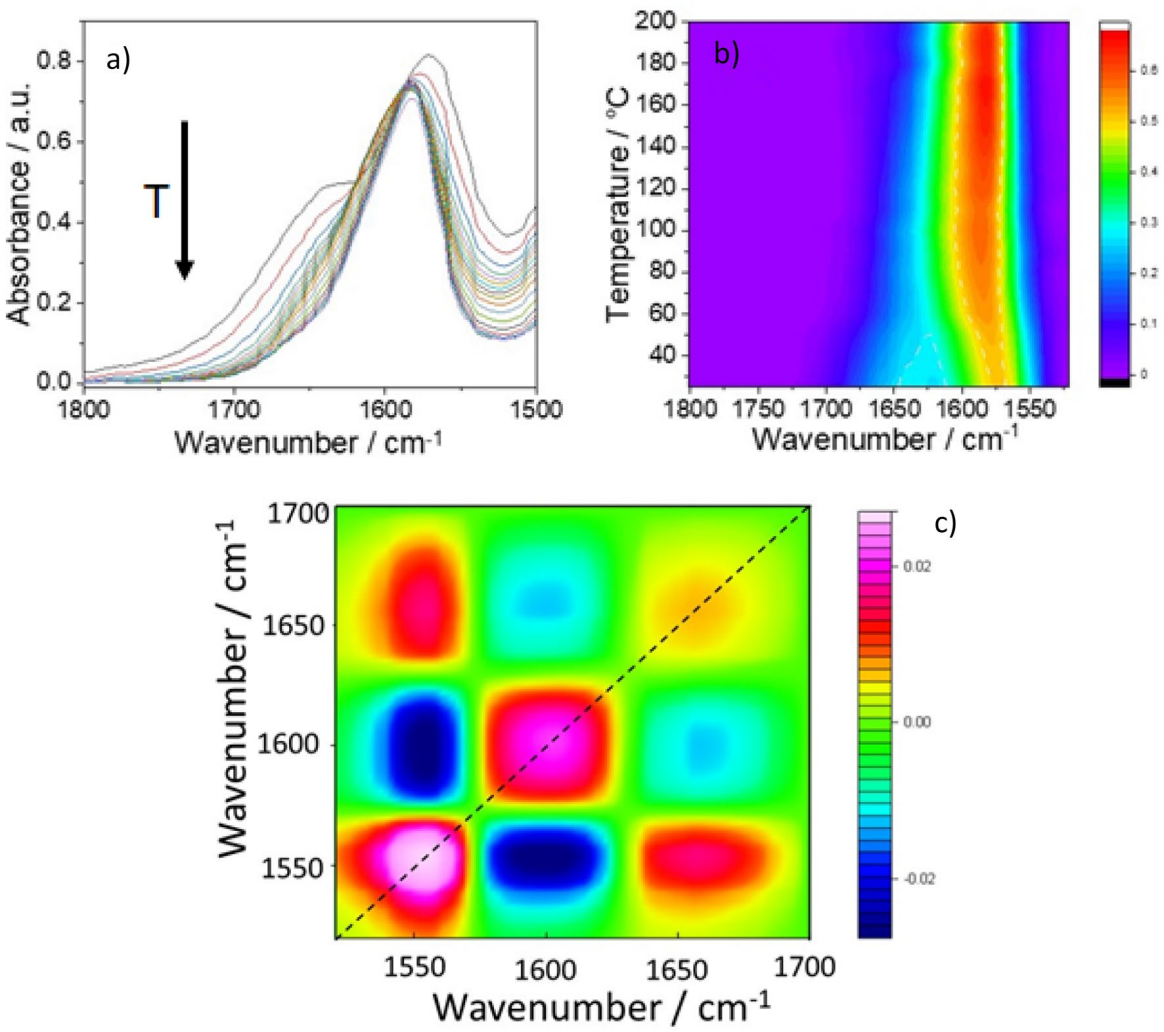
(**a**) FTIR absorption spectra of the pH 13 samples obtained by HT at 200 °C in the 1800–1200 cm^−1^ range and recorded in situ at increasing temperatures, from 25 up to 200 °C. (**b**) 3D map of the FTIR spectra, wavenumber (X-axis)–temperature (Y-axis)–intensity (false color scale). (**c**) 2D IR correlation asynchronous spectra; the intensity is shown in a false color scale.

The spectra are characterized by the signals of the amide I (C = O v_sym_) 1625–1700 cm^−1^, and amide II (in-plane deformation mode of NH groups) 1530–1570 cm^−1^ regions^20^. The rise of the amide I and II bands indicate the progress of the polycondensation through the formation of amide bonds. The polymeric form of l-lysine at the end of the hydrothermal process shows different structures that depend on the ph. The spectra of the “alkaline” samples (precursors aqueous solutions at pH 9.7 and 13) are different from the “acid” and “neutral” one (pH 2.5 and 7.3); they have infrared bands (around 1640 and 1670 cm^−1^) that reveal the presence of conformational structures^21^. To investigate more in detail the presence of organized structures we have performed an in-situ FTIR analysis in temperature. The presence of conformational structures, such as α-helix and β-strand, should be revealed by the changes of the spectra with the temperature during the denaturation process. Figure 8a shows the FTIR spectra of the sample at pH 13, as a function of the temperature from 25 up to 200 °C in the amide I and II regions (1800–1550 cm^−1^). In Fig. 8b, the same spectra are shown as a 3D map (x-wavenumber–y-temperature–intensity in false color scale). The FTIR spectra exhibit two absorption bands. one at 1570 cm^−1^ assigned to the residual carboxylic –COO^−^ in the deprotonated form and a shoulder at 1636 cm^−1^ assigned to amide I. The latter suggests the presence of β-strand conformations into the poly-l-lysine secondary structure. With the increase of temperature, the main band decreases in intensity, shifting to higher wavenumbers (from 1570 to 1582 cm^−1^) and sharpens. The trend of these two FTIR bands indicates the presence of strong hydrogen bonds that progressively decrease with the temperature increasing. The disappearance of the shoulder at 1636 cm^−1^ indicates that the weakening of the H-bonds progressively loses the intermolecular β-strand conformations leaving intact only the intramolecular one.

Figure 8c shows the 2D synchronous correlation IR map. Along the diagonal, three autopeaks of the same sign can be recognized at 1560, 1600, and 1650 cm^−1^. The false color scale gives the spectra intensity variation as a function of the change in T (∆T = T_200_ – T_25_). Because the three autopeaks have the same sign, this means that with the increase of the external variable, the temperature, they all increase in intensity. Interestingly, in the 1700–1500 cm^−1^ region, the correlation map reveals the presence of three components that change with the temperature. The correlation map allows achieving an unambiguous observation of the third component at higher wavenumbers. This observation enables a tentative assignment of the component at higher wavenumbers to the α-helix conformation. The different intensities of the two bands suggest that the β-strand is the predominant conformation that l-lysine assumes when it polymerizes starting from a high pH solution. The α-helix is more difficult to form and should be present to a lesser extent.

**Figure 9:**
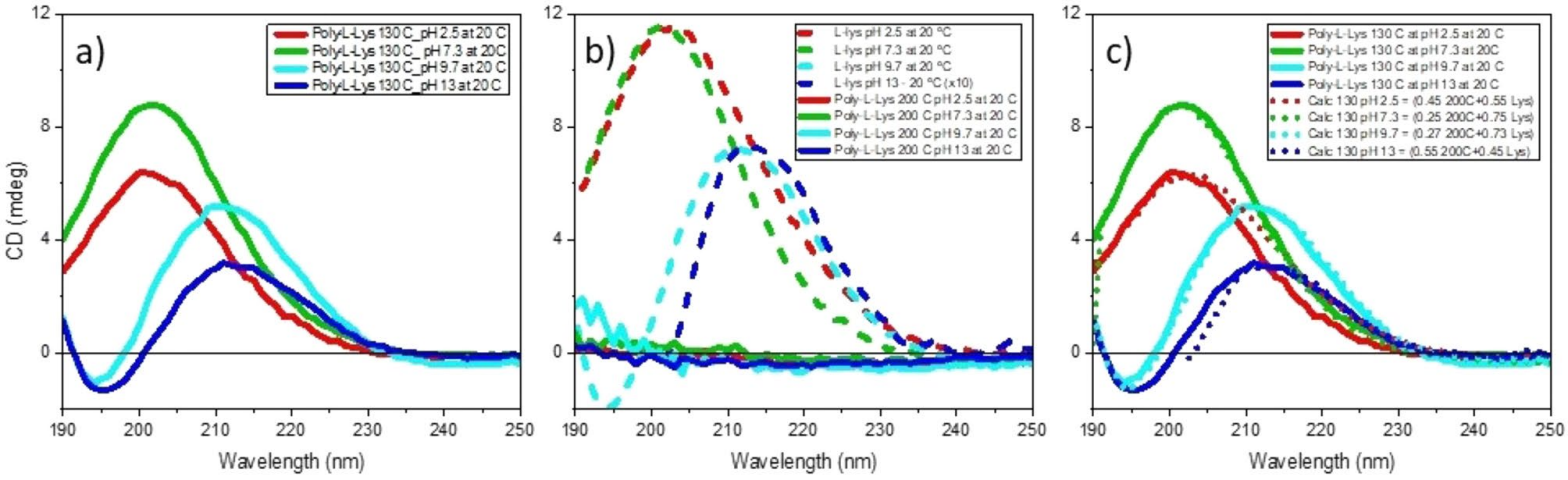
(**a**) CD spectra of HT-130 °C poly-l-lysine prepared from l-lysine aqueous solutions at different pHs (2.5, green line; 7.3, sky blue line; 9.7, red line; blue, 13). The samples have been measured at 20 °C. (**b**) CD spectra of HT-200 °C poly-l-lysine prepared from l-lysine aqueous solutions at different pHs (2.5, green line; 7.3, sky blue line; 9.7, red line; blue, 13). The samples have been measured at 20 °C. The CD spectra of pure l-lysine in aqueous solutions at different pH are the dashed lines. (**c**) CD spectra of HT-130 °C poly-l-lysine from the figure a, overlapped to the simulated CD spectra shown as dotted lines. The simulations have been performed by adding different fractions of HT-200 °C PLL (see Fig. 10b) to the corresponding l-lysine aqueous solutions. The CD spectra of l-lysine at pH 13 is normalised because it has been measured with 0.1 cm path length instead of 0.01 cm employed in the other measures.

Circular dichroism (CD) analysis has been employed to support the FTIR findings and get a more detailed analysis of the spatial arrangement in the different PLL structures. (Fig. 9). Figure 9a shows the CD spectra of HT-130 °C PLL prepared from different pHs and measured at 20 °C. The CD spectra measured for the samples from lower pHs (2.5 and 7.3) show positive band maxima at 200 nm that red shift to 210 nm for pH 9.7 and 13 PLLs (Fig. 9a). These spectra are not resembling any of the conformations that a-poly-l-lysine is capable of adopting, such as α-helix, β-strand, poly proline of type II (PII), and unordered conformations^22^. On the other hand, in the case of an ε-poly-l-lysine polymer, the amide bonds are isolated, quite far from each in the structure, and do not couple like in the α-poly-l-lysine generating the characteristic canonical CD shapes of secondary structure protein conformations.

**Figure 10:**
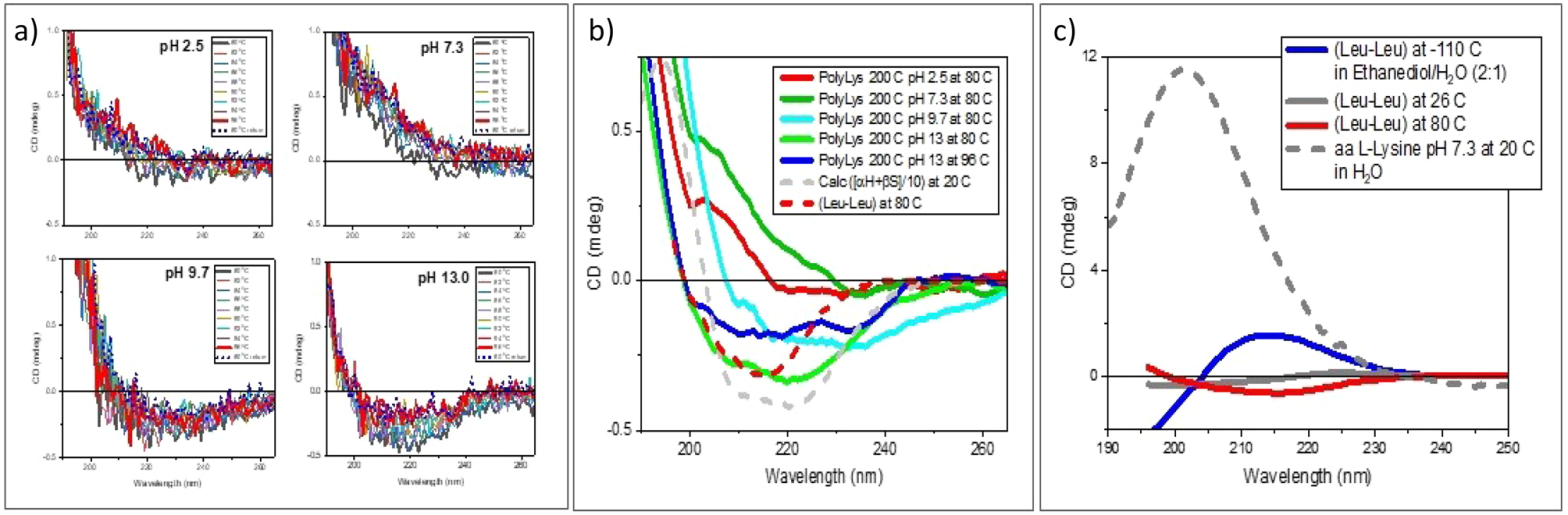
(**a**) CD spectra of HT-200 °C l-lysine in water at four different pHs (2.5, 7.3, 9.7 and 13) as a function of the temperature between 80 and 96 °C (measuring steps, 2 °C in heating and cooling). (**b**) Selected smoothed spectra from the figure a overlapped to a CD spectrum (reduced by 10 folds) of a protein with a secondary structures formed by 16% of α-Helix, 33% β-Strand, 21% Turn, and 30% Unordered. The simulation (grey dashed line) has been performed using a CONTINN algorithm^11,23,24^ of B23 CDApps^10^ and Leucyl–Leucine (Leu–Leu) (dashed red). (**c**) CD spectra of Leucyl–Leucine in ethanediol/H_2_O (2:1) at − 110 °C (blue), 26 °C (grey) 80 °C (red) and l-lysine at pH 7.3 at 20 °C in H_2_O (dashed grey)^9^.

The HT-200 °C PLLs, measured at 20 °C, show, however, featureless CD spectra of very negligible intensity for all the pHs (Fig. 9b), indicating spectral cancellation. This effect is shown in Fig. 10c for the dipeptide Leucyl-Leucine at 20 °C used as model. Conformations of the opposite sign, like those assigned to left-handed extended polyproline of type II at − 100 °C and the β-strand contribution at 80 °C^23^, produce spectral cancellation. α-poly-l-lysine^24^ and oligopeptides^22^ in the same temperature range also show a similar effect.

The HT-130 °C PLLs spectra are qualitatively similar, besides some differences in magnitude, to those of pristine l-lysine in aqueous solutions at the different pHs (Fig. 9b). The simulated spectra, calculated using a linear combination of various fractions (Table S1) of HT-200 °C PLL and pure l-lysine at the corresponding pHs, correlate well with the measured spectra of HT-130 °C PLL (Fig. 9c). The CD spectral contribution from the l-lysine fractions indicates that part of the amino acid does not polymerize. On the contrary, the HT-200 °C l-lysine fraction can be seen as the degree of polymerization that generates the amide bond chromophore. At the same time, large portions of assigned unreacted amino acid l-lysine, from 25 to 55%, are pH-dependent (Table S1).

In summary, HT-130 °C l-lysine at pH 2.5 and 13 shows around 50% of polymerization. However, at pH 7.3 and 9.7, the polymerization decreases to about 25% (Fig. 9c and Table S1). For HT-130 °C PLL, polymerization mainly goes through ε-NH_2_ of the amino acid side-chain. The CD spectral similarities between the pure l-lysine and the HT-130 °C samples under the same pH conditions well support this hypothesis, in agreement with the interpretation of the FTIR and NMR data. Such polymerization cannot be obtained in significant amounts if it goes through the α-NH_2_ amines. In fact, the CD spectral features of secondary structure conformations in canonical proteins should be at least an order of intensity magnitude bigger than those of pristine l-lysine under the same pH conditions (Fig. 9b and Fig. SI4).

To assess the type of polymerization, the effect of the temperature on the CD spectra recorded between 80 and 96 °C using a 2 °C step has been studied on both HT-130 °C and HT-200 °C series of samples under the different pH conditions (Fig. 10a).

For each one of the HT-130 °C PLL samples, the CD spectra acquired between 80 and 96 °C show very little changes Figure SI5) compared to those measured at 20 °C for the corresponding pHs (Fig. 9b).

However, this was not the case for all HT-200 °C samples measured in the 80–96 °C range. The qualitative and quantitative similarity with the CD spectra of dipeptide l-leucyl-l-leucine (Leu–Leu) at 80 °C is consistent with the growth of an ε-poly-l-lysine polymer that forms from high pH l-lysine solutions (Fig. 10b), in agreement with the NMR results (Fig. 5). These spectra have been then compared with a simulated spectrum of a protein (overall intensity reduced by 10 times) having a structural conformation given by different components, 16% of α-Helix, 33% β-Strand, 21% Turn, and 30% Unordered secondary structure (Fig. 10b, grey dashed line). This comparison reiterates the view that no or very small contributions from α-poly-l-lysine are present in the HT-200 °C PLL samples.

In the HT-130 °C PLLs, the UV denaturation assay has shown that the l-lysine polymers are stable with very small changes that can be compared to those observed in samples perturbed by thermal denaturation (data not shown).

**Figure 11:**
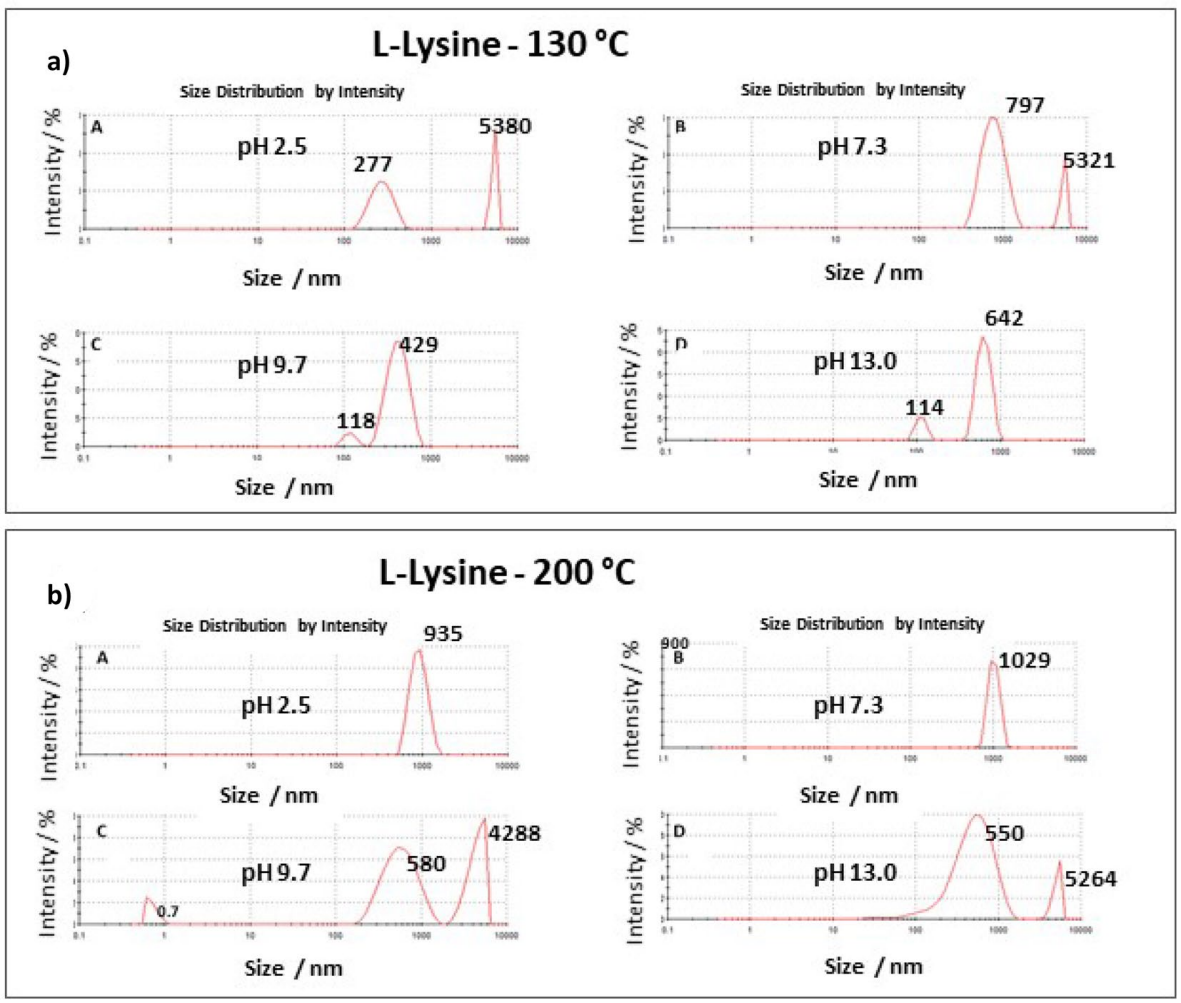
Size distribution by intensity of Dynamic Light Scattering of HTT-130 °C PLL (**a**) and HTT-200 °C (**b**) using as precursors the l-lysine aqueous solutions at pH 2.5, 7.3, 9.7 and 13.

Dynamic Light Scattering (DLS) measurements performed to assess the size and distribution of the components in each sample revealed a large range of assemblies that changed according to the pH and polymerization temperature (Fig. 11). The DLS data show that polymerization at HT-200 °C forms a more homogenous ε-poly-l-lysine structure with 100% population from volume vs. size measurements. The hydrodynamic radius is ~ 1000 nm at pH 2.5 and 7.3, while a bimodal distribution is observed at pH 9.7. At pH 13, PLL has an almost monomodal distribution, with around 90% of the particles having an average radius of 550 nm. On the other hand, the HTT performed at 130 °C yielded heterogeneous ε-poly-l-lysine with various sizes (Table S2).

The experimental data have indicated that the control of the architecture of l-lysine-based polymers is directly connected with the reactivity of the two amino groups present in the amino acid. As we have previously pointed out, the reactivity of the ε-NH_2_ groups is greater than those of α-NH_2_ and it is generally observed a preferential polymerization through the amines in ε. Therefore, the possibility of obtaining cross-linked three-dimensional structures, such as dendrimers or hyperbranched polymers, depends on the ability to modulate the reactivity of the two different amines. For this reason, most of the syntheses designed to obtain cross-linked polymers use protective groups that can be reversible or temporary. The comparative analysis of the structures obtained after hydrothermal synthesis, however, has shown how the polymer architecture can be modulated, albeit within certain limits, which depend on the pH of the precursor solution. To better understand how the protonation state of l-lysine affects the polymerization (Fig. 1), it is important to estimate the ratio of the protonated form versus the neutral form of the amino groups. This ratio is in correlation with the linear versus branched/dendrimeric growth. The Henderson–Hasselbach Eq. (1) gives the amount of ammonium. In addition, the equation allows for evaluating the ratio between the protonated and neutral state of l-lysine as a function of the pH of the solution and the dissociation constant of the corresponding acidic form (*K*_*a*_).

1 $$pH=p{K}_{a}+log\frac{[\alpha {NH}_{2}]}{[\alpha {NH}_{3}^{+}]}$$

Equation (1) can be rearranged in the following form:

2 $$\frac{\left[\alpha {NH}_{3}^{+}\right]}{\left[{\alpha NH}_{2}\right]}={10}^{p{K}_{a}-pH}$$

Equation (2) directly calculates the ratio of the acidic species (–NH_3_^+^) and its conjugated base (–NH_2_) using the anti-logarithm of the difference between the *pK*_*a*_ of the species and the actual pH of the solution.

In summary, at pH 13 the only specie in the solution is the negatively charged (**Lys**^**−**^) that is responsible of the higher reactivity of the ε-nitrogen and leads to the formation of a linear polymer as the most abundant product. Dropping the pH above 9.7 (excess of carboxylate ion), the third dissociation constant, *pK*_*a3*_ associated with the ε-amino group and equal to 10.67 need to be considered. Equation (2) allows to estimate that only 10% of the lysine ε-amino groups are in a protonated state (**Lys**^**+**^) while 90% are either neutral (**Lys**^**0**^) or negatively charged (carboxylate group) (**Lys**^**−**^) (Fig. 1). When considering the two amino groups in their neutral form, the lone pair of the ε-nitrogen is slightly affected by the nearby presence of the negatively charged carboxylate groups, which reduces its availability, thus hampering its nucleophilicity. On the other hand, the ε-nitrogen does not have any species around that can hinder the availability of its lone pair and hence its capability of a nucleophilic attack toward a carboxylate group. Under this condition, the higher nucleophilicity of α-nitrogen *pK*_*a3*_ = 10.67 compared to the *pK*_*a2*_ = 9.16 favours the amidation, promoting the formation of linear polymers (through ε-nitrogen) with little branching (through α-nitrogen). This peculiar trend is observed because the higher reactivity of the ε-NH_2_ is reduced by the partial protonation and overtaken by the higher abundancy of the α-NH_2_ that can react forming either branching or dendrimers.

With a pH below 7.5 (excess of ammonium ion), not all the l-lysine residues are fully protonated. In fact, while the ε-nitrogen is predominantly protonated for pH lower than 9.16 (*pK*_*a2*_), the amount of α-nitrogen in its ammonium form depends on the exact pH affecting its reactivity in the amidation process. Considering the dissociation equilibrium of the α-NH_3_^+^ into α-NH_2_ and that the corresponding *pK*_*a*_ is equal to 9.16, it is possible to calculate the relative ratio of the protonated versus the neutral form of the α-amino group. For pH 7.3, the ratio (α-NH_3_^+^/α-NH_2_) is about 72:1 indicating the linear polymerisation being highly unfavoured due to the lack of neutral ε-amino groups. Although most of the α-amino groups are in the protonated form, a small percentage of 1.4% is still in the neutral form, α-NH_2_, which can form branched/dendrimeric structures. This dramatic increase in the ratio of the protonated vs. a neutral form of the α-NH_2_ explains why at pH 7.3 the polymerization proceeds exclusively through highly cross-linked structures. At pH 2.5, the ratio between protonated and neutral species reaches 6.6 × 10^6^, meaning that in solution, all the nitrogens are protonated making the formation of polymeric structures based on amidation reactions impossible. The ^1^H-NMR analysis of the HTT-200 °C pH 2.5 samples that show mostly unreacted l-lysine monomers supports these findings. This study allows opening a new perspective in the synthesis of homo and polypeptides based on l-lysine with the possibility of modulating their structure controlling the pH and temperature of the precursor amino acid solution.

## Conclusions

In the present work, a systematic study of the hydrothermal synthesis of poly-l-lysine, starting from aqueous solutions with different pHs, has been realized. Furthermore, the study aims to understand how to modulate polylysine structural and physical–chemical properties by controlling the overall charge of the precursor.

The structural analysis of the polylysine obtained after a hydrothermal treatment at 200 °C has shown significant differences in the homopeptide architecture as a function of the pH. The polylysine processed from low pH is a hyperbranched cross-linked polymer. In contrast, a high pH allows forming of linear structures due to the higher reactivity of the e-amino groups. The correspondence between the pH of the precursor solution and the final polylysine structure indicates the dependence on the protonation state of l-lysine. The protonation of both the ε and α-amine groups favors the formation of an interconnected structure (pH 2.5 and 7.3); on the contrary if the overall l-lysine charge is 0 or − 1, ε-poly-l-lysine forms (pH 9.7 and 13). The linear polylysine shows a conformational spatial arrangement consistent with e-amide bonds. The final polymers emit in the blue range due to the increasing number of intermolecular hydrogen bonds and the more efficient charge transfer responsible for the emission. Thus, the results show a close correlation between the charge state of l-lysine in solution and the polymeric species formed through amidation reactions. It is possible to preferentially obtain cross-linked or linear lysine polymers by modulating the pH of the starting solution. This finding opens the route to a fine design of homopolypeptides and polypeptides based on l-lysine.

## Supplementary Information

Supplementary Information.

## Data Availability

Original data will be available on request to the corresponding author.

## References

[1] Yang G, Ji J, Zhang S, Li G, Li B (2020). Colorimetric chiral discrimination of lysine enantiomers and configurable logic gate operation based on fluorescein-functionalized polydiacetylene vesicles. Anal. Methods.

[2] Lugasi L, Otis G, Oliel M, Margel S, Mastai Y (2022). Chirality of proteinoid nanoparticles made of lysine and phenylalanine. Polym. Adv. Technol..

[3] Stagi L, Malfatti L, Caboi F, Innocenzi P (2021). Thermal Induced polymerization of l-lysine forms branched particles with blue fluorescence. Macromol. Chem. Phys..

[4] Thompson M, Scholz C (2021). Highly branched polymers based on poly(amino acid)s for biomedical application. Nanomaterials.

[5] Cieślik-Boczula K (2017). Alpha-helix to beta-sheet transition in long-chain poly-l-lysine: Formation of alpha-helical fibrils by poly-l-lysine. Biochimie.

[6] Stagi L (2022). At the root of l-lysine emission in aqueous solutions. Spectrochim. Acta Part A..

[7] Cossu FL, Poddighe M, Stagi L, Anedda R, Innocenzi P (2022). The birth of fluorescence from thermally polymerized glycine. Macromol. Chem. Phys..

[8] Krȩżel A, Bal W (2004). A formula for correlating pKa values determined in D_2_O and H_2_O. J. Inorg. Biochem..

[9] Siligardi G (1991). The Analysis of Molecular Configuration and Conformation of Biologically Important Molecules using Electronic UV and IR Circular Dichroism Spectroscopy.

[10] Hussain R (2015). CDApps: Integrated software for experimental planning and data processing at beamline B23 diamond light source. J. Synchrotron Radiat..

[11] Provencher SW, Gloeckner J (1981). Estimation of globular protein secondary structure from circular dichroism. Biochemistry.

[12] Homchaudhuri L, Swaminathan R (2004). Near ultraviolet absorption arising from lysine residues in close proximity: A probe to monitor protein unfolding and aggregation in lysine-rich proteins. Bull. Chem. Soc. Jpn..

[13] Homchaudhuri L, Swaminathan R (2001). Novel absorption and fluorescence characteristics of l-lysine. Chem. Lett..

[14] Nolting D (2007). pH-induced protonation of lysine in aqueous solution causes chemical shifts in X-ray photoelectron spectroscopy. J. Am. Chem. Soc..

[15] Kitadai N, Yokoyama T, Nakashima S (2009). ATR-IR spectroscopic study of l-lysine adsorption on amorphous silica. J. Colloid Interface Sci..

[16] Ustunol IB, Gonzalez-Pech NI, Grassian VH (2019). pH-dependent adsorption of α-amino acids, lysine, glutamic acid, serine and glycine, on TiO2 nanoparticle surfaces. J. Colloid Interface Sci..

[17] Stagi L (2021). Effective SARS-CoV-2 antiviral activity of hyperbranched polylysine nanopolymers. Nanoscale.

[18] Scholl M, Nguyen TQ, Bruchmann B, Klok H-A (2007). The thermal polymerization of amino acids revisited: Synthesis and structural characterization of hyperbranched polymers from l-lysine. J. Polym. Sci. A..

[19] Kitadai N, Yokoyama T, Nakashima S (2009). In situ ATR-IR investigation of l-lysine adsorption on montmorillonite. J. Colloid Interface Sci..

[20] Rozenberg M, Shoham G (2007). FTIR spectra of solid poly-l-lysine in the stretching NH mode range. Biophys. Chem..

[21] Yang H, Yang S, Kong J, Dong A, Yu S (2015). Obtaining information about protein secondary structures in aqueous solution using Fourier transform IR spectroscopy. Nat. Protoc..

[22] Siligardi G, Drake AF (1995). The importance of extended conformations and in particular, the PII conformation for the molecular recognition of peptides. Biopolymers.

[23] Sreerama N, Woody RWB (2004). Computation and analysis of protein circular dichroism spectra. Numer. Comput. Methods D.

[24] van Stokkum IHM, Spoelder HJW, Bloemendal M, van Grondelle R, Groen FCA (1990). Estimation of protein secondary structure and error analysis from circular dichroism spectra. Anal. Biochem..

