# Supplementary material for: Modulating the poly-l-lysine structure through the control of the protonation–deprotonation state of l-lysine
Source: Sci Rep. 2022 Nov 16;12:19719. doi: 10.1038/s41598-022-24109-5 (PMC9668811; doi:10.1038/s41598-022-24109-5)
Supplement: Supplementary file 1 — Supplementary Information. [file 41598_2022_24109_MOESM1_ESM.docx]

**SUPPLEMENTARY INFORMATION**

for

**Modulating the poly-L-lysine structure through the control of the protonation-deprotonation state of L-lysine**

Luigi Stagi^1^, Martina Sini^2^, Davide Carboni^2^, Roberto Anedda^3^, Giuliano Siligardi^4^, Tiberiu-Marius Gianga^4^, Rohanah Hussain^4^, Plinio Innocenzi^2,5^*

^1^Department of Chemistry, Physics, Mathematics and Natural Sciences, University of Sassari, 07100 Sassari, Italy

^2^Laboratory of Materials Science and Nanotechnology (LMNT), Department of Biomedical Sciences, CR-INSTM, University of Sassari, 07100 Sassari, Italy

^3^Porto Conte Ricerche. Strada Provinciale 55, Porto Conte Capo Caccia, km. 8,400, Alghero (SS), 07041, Italy

^4^Diamond Light Source Ltd., Harwell Science and Innovation Campus, Didcot, United Kingdom

^5^ College of Science, Department of Chemistry. United Arab Emirates University. Al Ain. United Arab Emirates

*Corresponding authors: [plinio@uniss.it](mailto:plinio@uniss.it);


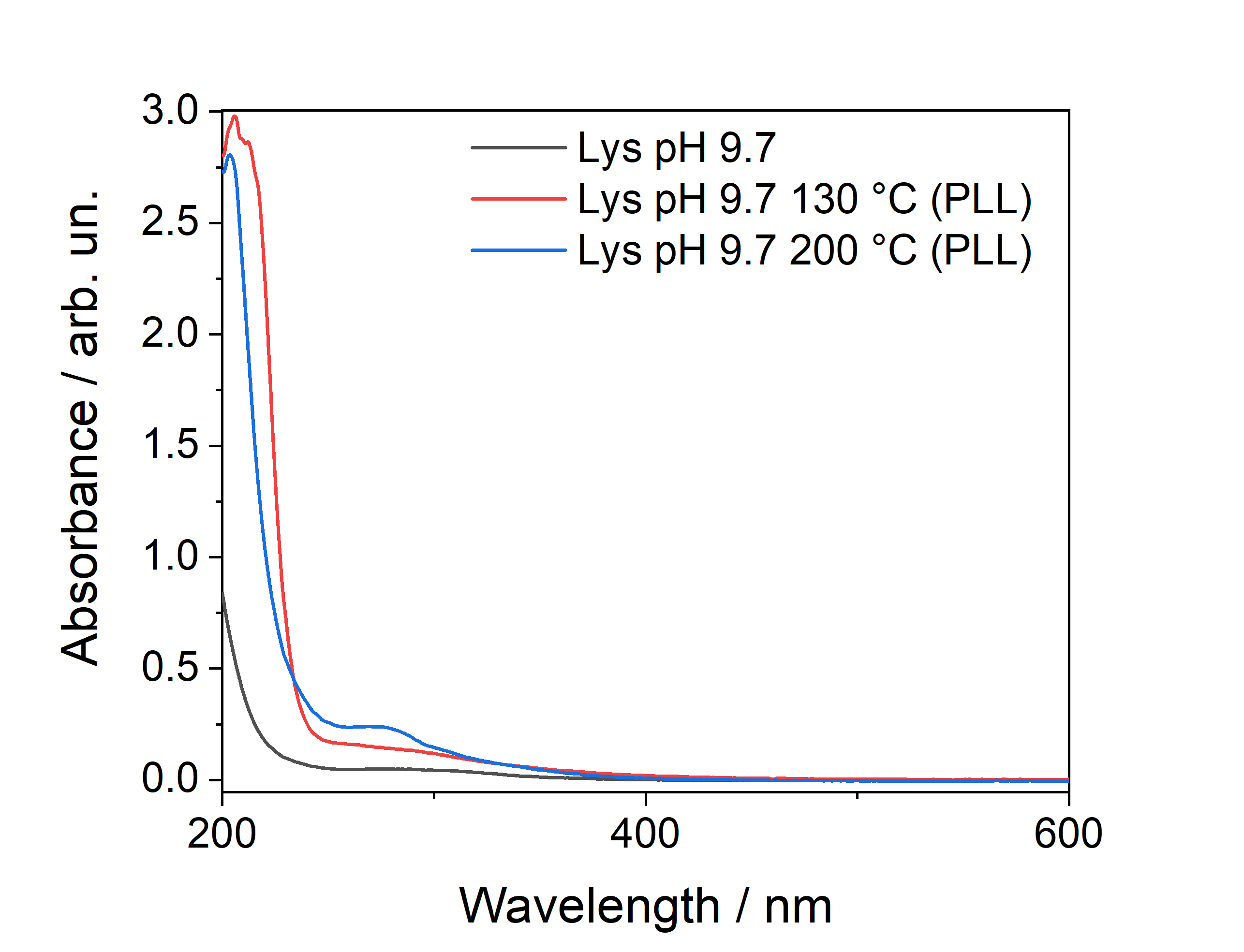


**Figure SI1**. UV-Vis spectra of an aqueous solution of L-lysine at pH 9.7 and polylysine in aqueous solutions obtained from the samples hydothermally treated at 130 and 200 °C. The precursor employed for HT was the aqueous solution of L-lysine at pH 9.7.


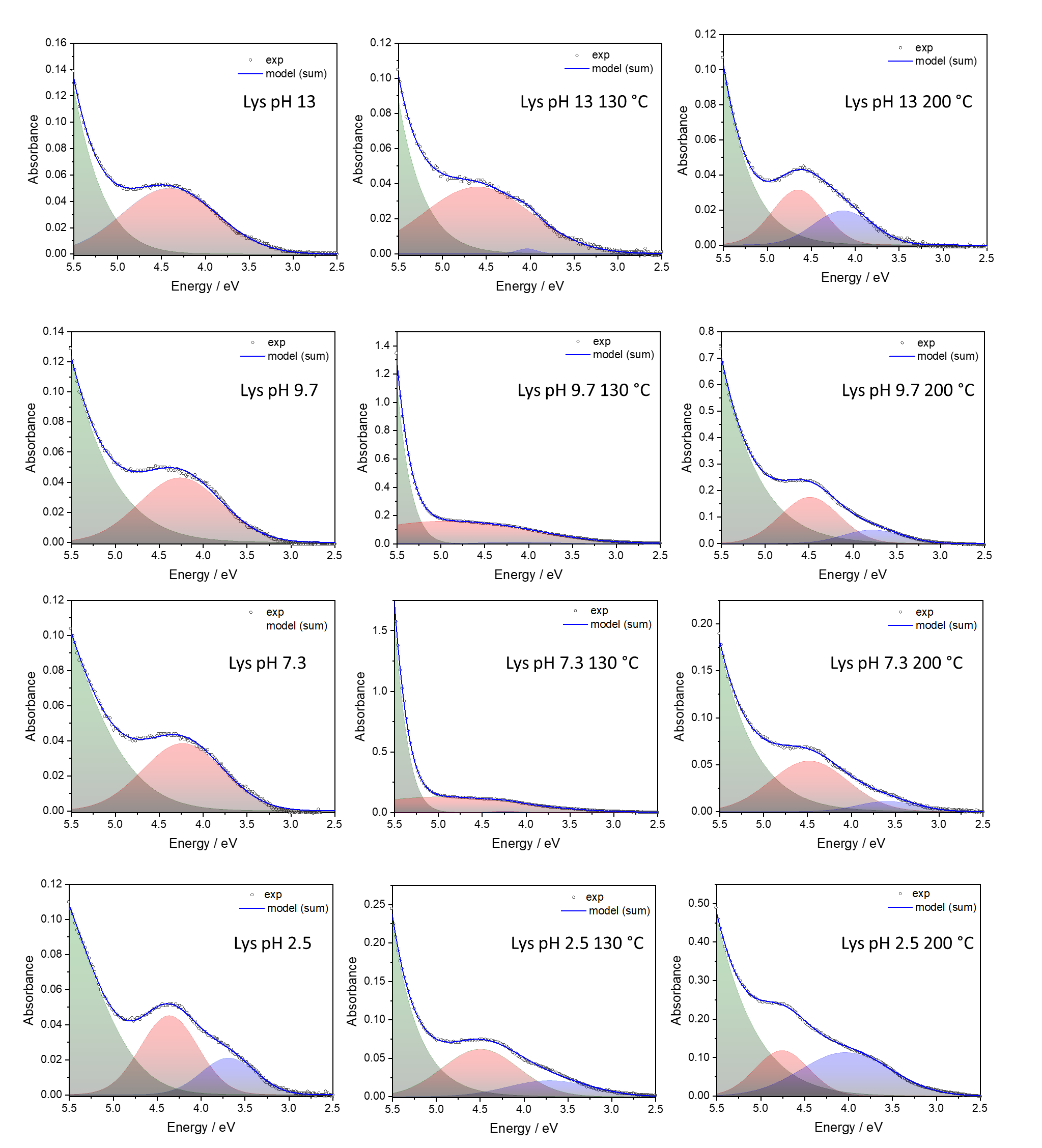


**Figure SI2**. Deconvolution with three components of the UV-Vis absorption spectra in the 5.5 – 2.5 eV range. The components are represented by green (UV component), red (4.5-4.3 eV) and blue (4.0-3.4 eV) curves. The experimental curve is represented by dot line and the fitting model by the blue curve.

| **Simulated spectra from linear combination of (A+B)** | **A**  ***(CD spectra of HT-200°C PLL)*** | **B**  ***(CD spectra of L-lysine at the corresponding pHs)*** |
| --- | --- | --- |
| *HT-130°C at pH 2.5* | 45% | 55% |
| *HT-130°C at pH 7.3* | 25% | 75% |
| *HT-130°C at pH 9.7* | 27% | 73% |
| *HT-130 °C at pH 13* | 55% | 45% |

**Table S1**. To simulate the spectra of HT-130°C PLL, have been used different values (reported in %) of HT 200°C PLL (column A) and L-lysine at the corresponding pHs (column B). The calculate spectra are a linear combination of spectra A with spectra B as shown in **Figure 9c**.

a)

| **Sample** | **Size / nm** | **Intensity / %** | **Size / nm** | **Volume / %** |
| --- | --- | --- | --- | --- |
| *HT-130°C – pH 2.5* | 276.6  5380 | 65.5  34.5 | 249.9  5397 | 42.8  57.2 |
| *HT-130°C- pH 7.3* | 797.3  5321 | 86.3  13.7 | 897  5328 | 90  10 |
| *HT-130°C – pH 9.7* | 429.4  118.1 | 92.7  7.3 | 458.4  115.8 | 94.1  5.9 |
| *HT-130°C – pH 13* | 642.1  113.6 | 85.7  14.3 | 682.9  110.3 | 92.7  7.3 |

b)

| **Sample** | **Size / nm** | **Intensity / %** | **Size / nm** | **Volume / %** |
| --- | --- | --- | --- | --- |
| *HT-200°C - pH 2.5* | 934.9 | 100 | 991.3 | 100 |
| *HT-200°C- pH 7.3* | 1029 | 100 | 1058 | 100 |
| *HT-200°C - pH 9.7* | 580  4288 | 64.1  35.9 | 756.3  3818 | 51.6  48.4 |
| *HT-200°C - pH 13* | 642.1  113.6 | 83.6  8.9  7.5 | 1086  81.61  5260 | 90.4  5.1  4.5 |

**Table S2**. Size distribution by intensity and volume of Dynamic Light Scattering data of L-lysine upon hydrothermal treatment at 130°C (a) and 200°C (a) using as precursors aqueous solutions at pH 2.5, 7.3, 9.7 and 13, respectively.

**Figure S3.** ^1^H NMR spectra of lysine (top) and hyperbranched polylysine nanopolymers (middle). Attribution of the chemical shifts (bottom) has been done following the data published in: Scholl, M., Nguyen, T. Q., Bruchmann, B., Klok, H.-A. The Thermal Polymerization of Amino Acids Revisited; Synthesis and Structural Characterization of Hyperbranched Polymers from L-Lysine. J. Polymer Sci.: Part A: Polymer Chem., 45, 5494–5508 (2007).

**H_6_** 2.67 ppm ε−CH_2_ group in a α-linear and terminal structural unit

**H_5_** 3.19 ppm ε-CH_2_ group next to an ***amide bond*** (dendritic and ε-linear structural unit)

**H_4_**  3.25 ppm α−CH protons of the ε-linear structural units

**H_3_** 3.33 ppm α−CH protons of the terminal structural units

**H_2_** 4.02 ppm α−CH protons of the α-linear structural units

**H_1_** 4.24 ppm α−CH protons of the dendritic protons

Figure S3 reproduced from SI in reference: Stagi, L. *et al.* Effective SARS-CoV-2 antiviral activity of hyperbranched polylysine nanopolymers. *Nanoscale* **13**, 16465–16476 (2021)


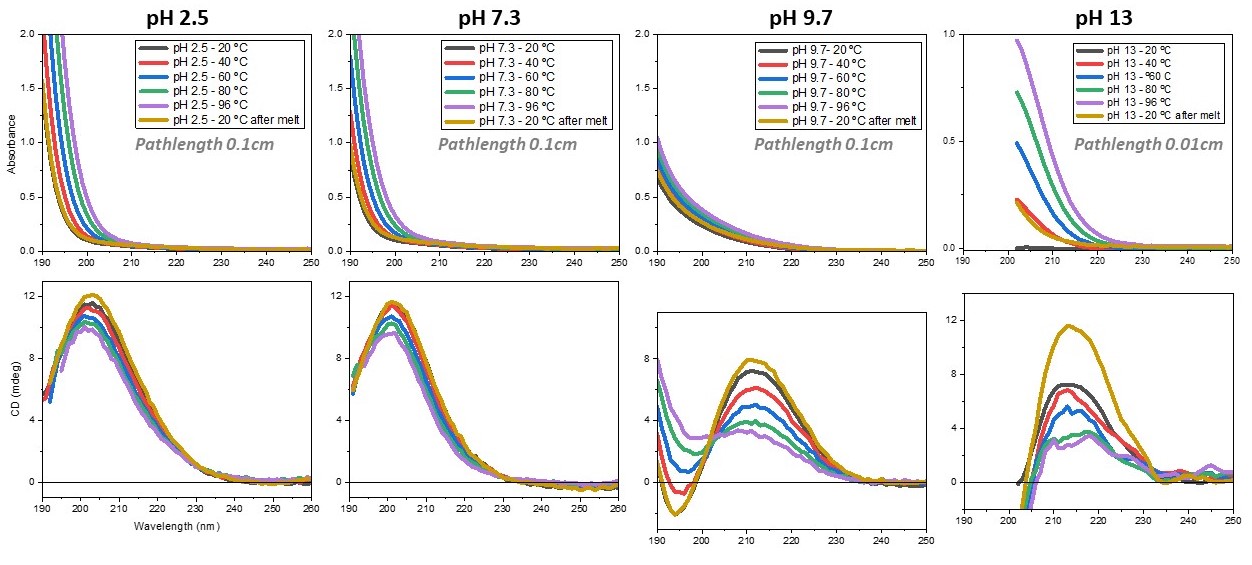


**Figure SI4.** CD spectra of aqueous L-lysine as function of temperature at pH 2.5, 7.3, 9.7 and 13, respectively.


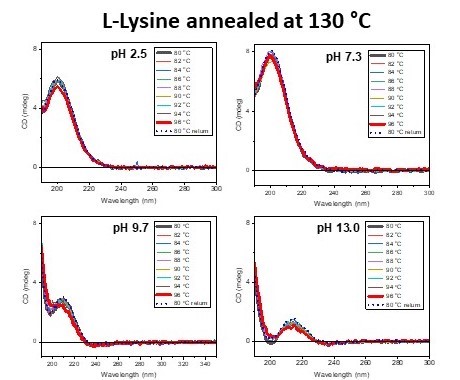


**Figure SI5.** CD spectra of L-lysine after HT at 130°C for four different pHs (2.5, 7.3, 9.7 and 13) and measured in the 80 to 96°C temperature range every 2°C and back to 80 °C.
